# Efficacy of artemether-lumefantrine as a treatment for uncomplicated *Plasmodium vivax* malaria in eastern Sudan

**DOI:** 10.1186/1475-2875-11-404

**Published:** 2012-12-05

**Authors:** Tajeldin M Abdallah, Abdel Aziem A Ali, Mohammed Bakri, Gasim I Gasim, Imad R Musa, Ishag Adam

**Affiliations:** 1 Faculty of Medicine, Kassala University, Kassala, Sudan; 2 Faculty of Medicine, University of Khartoum, P.O. Box 102, Khartoum, Sudan; 3 Faculty of Medicine, Qassim University, Qassim, Qassim, Kingdom of Saudi Arabia; 4 Buraidah Central Hospital, Buraidah, Kingdom of Saudi Arabia

**Keywords:** Efficacy, Artemether-lumefantrine, Malaria, P. vivax, Sudan.

## Abstract

**Background:**

Artemisinin-based combination therapy (ACT) is the treatment of choice for uncomplicated *Plasmodium falciparum* malaria in most areas of the world, where malaria is endemic, including Sudan. However, few published data are available on the use of ACT for treatment of *P. vivax* malaria.

**Methods:**

This study was conducted at a health centre in Kassala, eastern Sudan, from October to December 2011. Patients with uncomplicated *P. vivax* malaria received artemether-lumefantrine (AL) tablets (containing 20mg artemether and 120 mg lumefantrine) and were monitored for 28 days.

**Results:**

Out of the 43 cases enrolled in this study, 38 completed the 28-day follow-up. Their mean age was 25.1 years (SD: 1.5). On day 3 following AL treatment, all of the patients were afebrile and aparasitaemic. By day 28, all 38 patients exhibited adequate clinical and parasitological responses to AL treatment. The cure rate was 100% and 88.4% for the per protocol analysis andfor the intention to treat analysis, respectively. Mild adverse effects (nausea, vomiting, abdominal pain, dizziness and/or rash) that resolved spontaneously were observed in four (10.5%) of the patients.

**Conclusion:**

AL combination therapy was fully effective for treatment of *P. vivax* malaria in the study in eastern Sudan.

**Trial registration:**

Trial. Gov: NCT01625871

## Background

*Plasmodium vivax* infection is a major global health problem. This species of parasite has the broadest geographic distribution of the five malaria species known to infect humans [1]. There are about 2.5 billion people at risk of malaria and an estimated 80 to 300 million clinical cases of *P. vivax* annually [2,3]. Although *P. vivax* is mainly endemic in Southeast Asia and Latin America [3], it has recently been observed in Ethiopia and Sudan [4-8].

Malaria is a important health problem in Sudan, and in 2002, an estimated 9 million disease episodes and 44,000 deaths from the disease occurred [9]. The spread of multidrug-resistant *Plasmodium falciparum* malaria in Sudan [10,11] has led to adoption of artemisinin-based combination therapy (ACT), with artesunate–sulphadoxine–pyrimethamine (AS–SP) and artemether–lumefantrine (AL) becoming the recommended first- and second-line treatments for uncomplicated *P. falciparum* malaria, respectively.

Early diagnosis and effective treatment with an appropriate drug is one of the main components of the World Health Organization’s strategy to reduce malaria related mortality [12]. Episodes of *P. vivax* infection should prompt urgent treatment with effective anti-malarial medication [13]. While most malaria endemic countries have adopted ACT to reduce the risk of multidrug resistant strains of *P. falciparum* occurring [10], chloroquine remains the first-line treatment for *P. vivax* in most endemic countries. However, there is growing evidence that the efficacy of chloroquine against *P. vivax* is declining in many areas, especially Southeast Asia [14-16]. Hence, the use of ACT against both *P. vivax* and *P. falciparum* is preferred, especially in a country like Sudan where chloroquine is no longer registered or available [17]. The far-reaching adoption of ACT as an effective first-line therapy for *P. falciparum* has led to a closer examination of their role in the management of *P. vivax* malaria. AL is one such combination therapy that is widely used to treat uncomplicated *P. falciparum* malaria, and is the first-line treatment for *P. vivax* malaria according to the national malaria control programme in Sudan. However, there is no published data on the efficacy of AL for the treatment of *P. vivax* malaria in Sudan. Therefore, the efficacy of AL for the treatment of uncomplicated *P. vivax* malaria was investigated in Sudan. The study was conducted at a health centre in eastern Sudan, an area characterized by unstable malaria transmission [18]. Recently, however, the most severe form of *P. vivax* malarial disease has been observed in the area [8]. The study is of paramount importance because it aims to provide Sudanese health care professionals and public health planners with the fundamental data necessary for developing an effective programme for malaria control.

## Methods

**Figure 1:**
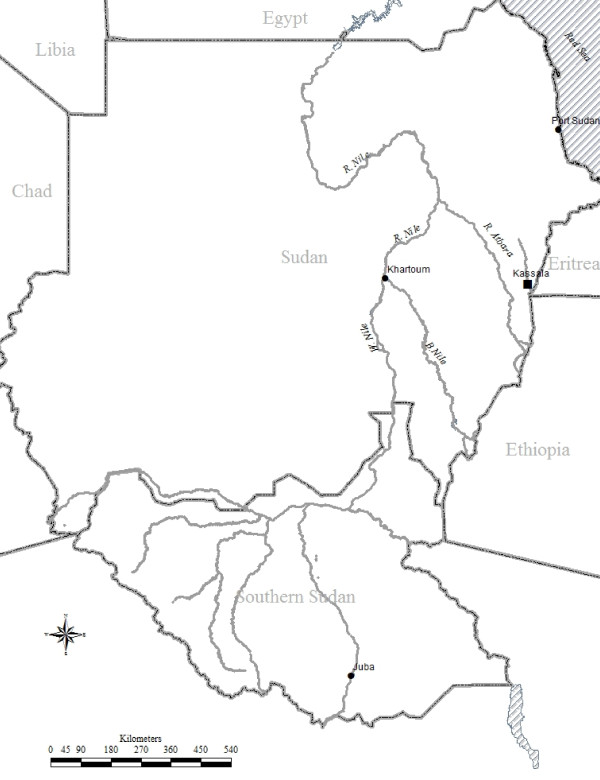
Map of Sudan.

This observational (without sample size calculation) clinical trial was conducted at the Fatima Eldeaig Health Centre in Kassala, eastern Sudan (located 600 km from Khartoum, Sudan) and took place from October to December 2011 (Figure 1). The World Health Organization (WHO) guidelines for assessment of the efficacy of anti-malarial drugs were adopted [19]. Febrile patients (axillary temperature ≥ 37.5°C) with confirmed blood films for *P. vivax* mono-infection and willing to participate in the study were enrolled. Those individuals with severe malnutrition, pregnancy, or with a history of allergy and/or intolerance to the drugs were excluded. Informed consent was obtained from all of the patients, or in the case of children, their guardians. Structured questionnaires were used together with socio-demographic characteristics, medical histories (including the duration of the illness) and physical findings.

Thick and thin blood smears were prepared and stained with 2% Giemsa solution for 30 minutes. Thick blood films were used for counting parasite numbers and for estimating parasite densities. Gametocytes were counted against 200 leukocytes, and parasite densities estimated using 8,000 white blood cells (WBCs)/μL of blood as the multiplier. Smears were considered negatives if no parasites could be seen in 100 high-power microscopic fields. Blood films were double checked (blind) by two independent examiners, and discordant results were agreed on after evaluating the evidence of each examiner. Parasite densities differing by more than 10% between the different microscopists were evaluated by a third microscopist, whose result was deemed to be final.

### Treatment

AL (Coartem®; Novartis, Basel, Switzerland) was administered over a three-day period. AL tablets contained 20 mg of artemether and 120 mg of lumefantrine. Patients were weighed and those corresponding to 5–14, 15–24, 25– 34 or >35 kg were each given six doses of AL (at 0, 8, 24, 36, 48 and 60 h), each dose consisted of one, two, three or four tablets, respectively. The morning dose was given at the health Centre under the direct supervision of a medical officer. Young children who could not swallow tablets were given suspensions created by crushing and dissolving their tablets in water. All patients were observed for vomiting for 1 h after each treatment. If vomiting occurred within 30 min of a dose, a second full dose was administered. If vomiting occurred 31–60 min after a dose, however, only half a dose was given. If vomiting occurred after a repeated (full or half) dose, the patient was given a quinine infusion and excluded from the study. These instructions were given to all of the patients (or guardian in the case of children) because the evening dose was given un-supervised. Patients also received unsupervised primaquine tablets (15 mg) daily for 14 days.

### Treatment outcomes

Patients were asked to return on days 1, 2, 3, 7, 14, 21 and 28 of the study, or at any other time if they felt unwell. During each visit, the participants’ temperatures were measured and blood smears were prepared and checked as described above. Treatment outcomes were classified according to WHO guidelines for evaluation of the therapeutic efficacy of antimalarial drugs in the treatment of uncomplicated malaria [19]. During follow-up, the patients were asked if they had any adverse effects that might have been expected from the treatment itself (nausea, vomiting, abdominal pain, dizziness and rash). These effects were considered to be AL-related if they had not been reported when the patient first presented for treatment at the Health Centre. The primary outcome was the cure rate, which was defined as the percentage of the patients completing follow-up that had adequate clinical and parasitological responses (ACPR) after 28 days. Secondary outcomes comprised the frequencies of early treatment failure, late clinical response, late parasitological response, and adverse events, and the time taken to reduce fever and clear parasitaemia. Early treatment failure was defined as the development of danger signs or severe malaria, in the presence of parasitaemia, on days 1, 2 and/or 3, a day-2 parasitaemia that was higher than the day-0, the presence of both fever and parasitaemia on day 3, and/or a day-3 parasitaemia that was 20% of the day-0 value. A late treatment failure was defined as the development of danger signs or severe malaria, in the presence of parasitaemia, after day 3, the presence of both fever and parasitaemia on or after day 4, and/or the presence of parasitaemia after day 7.

### Data analysis

Data were entered into a computer database and analysed using SPSS software (version 20, Chicago, IL, USA). Mean and SD were calculated for the variables presented. The intention-to-treat analysis included all enrolled patients who met the inclusion criteria and took at least one full dose of AL. Patients lost to follow-up or withdrawn from the study were considered to be treatment failures. The per protocol (PP) analysis of outcomes included data for patients who had completed the follow-up. Those patients lost to the follow-up or were withdrawn because of protocol violations were excluded from the PP analysis.

### Ethics

The current study received ethical approval from the Health Research Board at the Ministry of Health in Kassala state, eastern Sudan. The trial was registered at Trial. Gov: NCT01625871.

## Results

**Figure 2:**
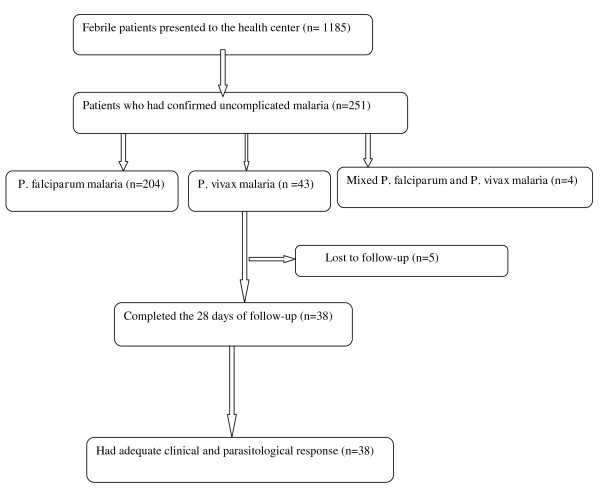
Patient enrollment and follow-up during the study.

During the study period, 1,185 febrile patients presented to the health centre, of which 251 had uncomplicated diagnoses of malaria confirmed. Out of these 251 patients, 204, 43, and four were uncomplicated *P. falciparum*, uncomplicated *P. vivax* and mixed *P. falciparum* and *P. vivax* infections, respectively (Figure 2). Therefore, the *P. falciparum: P. vivax* ratio was 4.7:1. Of the 43 enrolled patients with uncomplicated *P. vivax* malaria, 38 (88.37%) completed the 28 days follow-up, but the remaining 5 were lost to follow- up. The base line characteristics of the study participants are shown in Table 1. The range of the age, weight and parasitaemia was 4–60 years, 13–95 kg years, and 200–8,480.0 rings/μl, respectively. The majority (24, 63.2%) of these patients were male. Only one patient was aged less than five years (a 4-year old). Excluding those with gametocytes on day 0 (two patients), no new carriers were identified during the 28-day follow-up. All of the day 0 slides that were submitted to the National Laboratory at the National Malaria Control Programme for quality control were identified as containing the same species of malaria parasite (i.e., *P. vivax*).

**Table 1:** Baseline characteristics of patients treated with artemether-lumefantrine in eastern Sudan

**Variable**	**Mean (SD)**
Age in years	25.1 (1.5)
Weight in kg	50.2 (2.5)
Temperature °C	38.5 (0.6)
Duration of the illness in days prior to diagnosis	4.1 ( 2.4)
Geometric mean of asexual-stage parasitaemia per μL	3272.0 (2029.3)

On day 1 all patients were afebrile, but 2 (5.3%) of them were parasitaemic. By day 3, all patients were afebrile and aparasitaemic. All of the patients 38 (100%) responded to treatment with AL and had ACPR. The cure rate was 100% and 88.4% for the per protocol analysis and for the intention to treat analysis, respectively. Mild adverse effects (nausea, vomiting, abdominal pain, dizziness and/or rash) that resolved spontaneously were observed in four (10.5%) patients.

## Discussion

The current study showed full efficacy (per protocol analyses) of the AL combination for treatment of *P. vivax* in eastern Sudan. Consistent with a recent study, the present study showed that *P. vivax* is a health problem and that its ratio to *P. falciparum* has increased [8]. Previous studies showed that AL had a high efficacy (98.7%) for treatment of uncomplicated *P. falciparum* malaria in eastern Sudan as well as other regions of the country [20,21]. Interestingly, in neighbouring Ethiopia, a higher failure rate was observed (19%) when AL was compared with chloroquine for treatment of uncomplicated *P. vivax* malaria [4]. The high AL failure rate in Ethiopia was attributed to the need for patients to take the evening dose of the drug unsupervised [4]. In this context, it is noteworthy that the emergence of chloroquine-resistant *P. vivax* was first confirmed in Ethiopia [22]. The full efficacy of AL for the treatment of *P. vivax* malaria in the present study is consistent with other studies that showed a rapid clearance of *P. vivax* parasitaemia and fever and an overall high cure rate for AL treatment of *P. vivax* malaria [13,23].

Chloroquine remains the drug of choice for treating *P. vivax* infection according to WHO guidelines and it is still effective in most malaria endemic areas [24,25]. Unfortunately, substantial levels of chloroquine-resistant *P. vivax* parasites have been observed in South East Asia [26-28]. In addition, some studies have reported chloroquine treatment failure for *P. vivax* malaria in Africa [4,22,29]. Therefore, the idea of using a single first-line therapy that is effective against both *P. vivax* and *P. falciparum* is attractive, especially in settings where two malaria parasite species are co-endemic. ACT is highly effective against uncomplicated *P. falciparum* malaria and, as a consequence of high levels of chloroquine resistance, is now widely adopted as the first-line therapy in most malaria endemic countries, including Sudan [10]. Indeed, some countries such as Papua New Guinea and Indonesia have adopted ACT as the first-line therapy for *P. vivax* and *P. falciparum* malaria [16]. A policy whereby use of a unified first-line therapy based on ACT was implemented could have great public health value in that it would simplify treatment, management and logistics of malaria disease control. It should be mentioned that chloroquine is no longer registered or available for use in Sudan, so as to avoid the problem of *P. falciparum* infection being treated with chloroquine.

The limitations of this study concern its non-randomized character and small sample size; however, with a lack of published data about the efficacy of AL against *P. vivax* malaria, this study provides useful insight into AL treatment of *P. vivax* infection. Secondly, the 28-day follow-up of patients treated with AL is another limitation; a longer follow-up period could be advantageous as it would provide more information about AL treatment outcomes. Thirdly, in this study, patients were treated with primaquine without prior assessment of their G6PD enzyme levels, because G6PD enzyme tests were not available.

## Conclusion

Although this study did not detect any treatment failures during treatment of uncomplicated *P. vivax* malaria using AL in this area of Sudan.

## Competing interest

The authors declare that they have no competing interests.

## Authors' contributions

TM, GIG, IA designed the study, AAA, IRM and MB conducted the clinical work. All of the authors drafted and approved the manuscript.
